# Analysis and evaluation of the entropy indices of a static network structure

**DOI:** 10.1038/s41598-017-09475-9

**Published:** 2017-08-24

**Authors:** Meng Cai, Ying Cui, H. Eugene Stanley

**Affiliations:** 1 School of Economics and Management, Xidian University, Xi’an, 710071 China; 2 School of Mechano-Electronic Engineering, Xidian University, Xi’an, 710071 China; 3 Center for Polymer Studies and Department of Physics, Boston University, Boston, Massachusetts 02215 USA

## Abstract

Although degree distribution entropy (DDE), SD structure entropy (SDSE), Wu structure entropy (WSE) and FB structure entropy (FBSE) are four static network structure entropy indices widely used to quantify the heterogeneity of a complex network, previous studies have paid little attention to their differing abilities to describe network structure. We calculate these four structure entropies for four benchmark networks and compare the results by measuring the ability of each index to characterize network heterogeneity. We find that SDSE and FBSE more accurately characterize network heterogeneity than WSE and DDE. We also find that existing benchmark networks fail to distinguish SDSE and FBSE because they cannot discriminate local and global network heterogeneity. We solve this problem by proposing an evolving caveman network that reveals the differences between structure entropy indices by comparing the sensitivities during the network evolutionary process. Mathematical analysis and computational simulation both indicate that FBSE describes the global topology variation in the evolutionary process of a caveman network, and that the other three structure entropy indices reflect only local network heterogeneity. Our study offers an expansive view of the structural complexity of networks and expands our understanding of complex network behavior.

## Introduction

A complex system is composed of a large number of interdependent and interactive subsystems, and complex network research abstracts the interactive relationship among these subsystems. Each node in a complex network represents a unit of the system, and edges between nodes stand for the interactions among the units. In a complex network the complicated relationships among nodes involve topology, function, and dynamic behavior^1^. Being able to describe this network complexity is an essential goal and is a perennial hot spot in complex network research^2, 3^.

Entropy is one physical aspect of complex system structure and quantifying entropy enables us to better understand the structural complexity and randomness of networks. Measuring entropy is currently a tool in such fields as discrete mathematics, computer science, information theory, statistics, chemistry, biology and medicine^4, 5^. Entropy in network science is both macroscopic and microscopic. Microscopically it focuses on fundamental particles (nodes) and generates a relative entropy index. As described in ref. 6, node microscopic entropy is defined in terms of the interactions between nodes (information rate). Tutzauer uses microscopic node entropy to measure node centrality^7^. Macroscopically it emphasizes network heterogeneity and includes two approaches. The first uses the angle of randomness and defines the entropy of a topological structure in terms of ER random network formation^8^. The second focuses on “connection” distribution homogeneity to clarify the entropy of a heterogeneous static network structure. This takes three forms. The first uses the angle of network nodes in which Wu structure entropy (WSE) indicates the randomness of network nodes^9^. The second uses network edges in which the degree distribution entropy (DDE) indicates the randomness of network edges^10^. The third takes both nodes and edges into consideration, as in SD structure entropy (SDSE)^11^ and FB structure entropy (FBSE)^12^.

Because a complex network system has an idiosyncratic evolutionary mechanism and statistical properties, to understand and carry out research on its topological properties and dynamic complexity we need a model that can demonstrate the statistical properties of a real-world network^1^. Currently the most popular models include those of a regular network, a random network, a scale-free network and a small world network. The most important among the regular networks include the nearest-neighbor coupled network in which the degree of each node stays the same and the star network in which all nodes are connected to the central node and not to any other nodes. When used to test network entropy indices, these two benchmark network models have, respectively, the largest and smallest network heterogeneity^11, 12^. In addition to these two systems with a regular structure, there are a number of benchmark networks that model complex network structure. For example, the ER random network models randomness^13^, the BA scale-free network models the scale-free property^14^, the WS small-world network models small world characteristics^15^, and the caveman network models community structure^16^.

Current research has made little use of benchmark networks when comparing and evaluating different network entropies. Cai *et al*. compared the heterogeneity indices of scale-free complex networks and found that WSE more accurately describes heterogeneity indices than DDE^12^. Using theoretical analysis and simulation experimentation on regular, random, and scale-free networks, they also found that SDSE more accurately reflects the features of network structure than WSE and DDE^11^. Although current benchmark networks can be used to test entropy indices, they still exhibit drawbacks. When evaluating entropy indices they make little use of mathematical analysis, and they provide only local information (i.e., node degree or degree distribution). We must be able to control local information when we use entropy indices to determine differences in the global topological structure (the node connection mode) of networks. For example, although the ER random network, the WS small-world network, and the caveman network appear to be homogeneous networks with a similar or equivalent node degree, their topological structures vary substantially^1^.

Here we draw on previous studies and use benchmark network models to analyze and evaluate the differences among several entropy indices of static network structure and network heterogeneity. Because these benchmark networks cannot reveal the difference between local and global network heterogeneities, we introduce the caveman evolutionary network with a community structure and find that it can evaluate how differing entropy indices affect network heterogeneity.

## Results

### Simulation results of typical networks

We compare the DDE, WSE, SDSE, and FBSE using simulation tests on a nearest-neighbor coupled network, a star network, an ER random network, and a BA scale-free network. We carry out the simulation tests on a PC with an Intel^R^Core^TM^ 2 2.40 GHz processor in a Matlab 7.0 environment. We calculate every entropy indicator 1000 times and take the average. Note that in all numerical entropy calculations we use the natural logarithm, and this allow us to compare the different network entropy indices.

**Figure 1:**
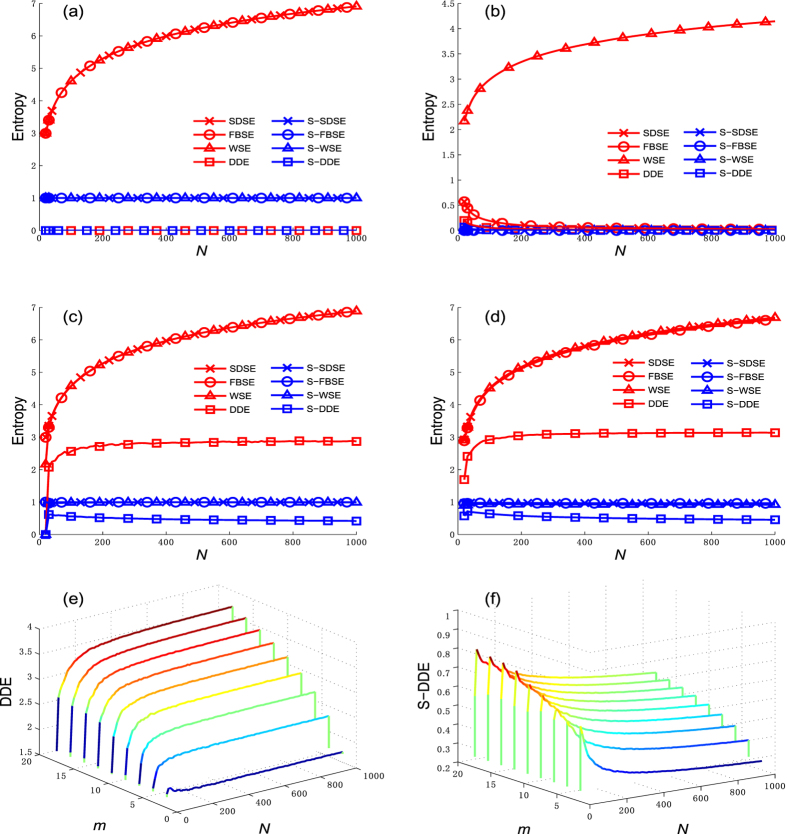
Different structure entropies of typical networks. S-SDSE, S-FBSE, S-WSE and S-DDE represent standardized SDSE, FBSE, WSE and DDE, respectively. (**a**) nearest–neighbor coupled network, (**b**) star network, (**c**) ER random network, (**d**) BA scale-free network, (**e**) DDE of BA scale–free network, (**f**) S–DDE of BA scale–free network.

Figure 1 shows the entropy values for four types of network. To eliminate the effect of network size, we define the standardized entropy index to be $\bar{H}=\frac{H-{H}_{min}}{{H}_{max}-{H}_{min}}\in [0,1]$. The curve of the BA scale-free network is of the entropy values during the evolving process in accordance with ref. 17, and the final network size is 1000. The curves of nearest-neighbor coupled network and the star network are of the entropy values for different network sizes. The ER random network is constructed in accordance with the network size and density of the BA scale-free network. We find that SDSE, FBSE, and WSE exhibit small changes as the growth rate *m* in the BA scale-free network changes (there are small increases in entropy as *m* increases). Figure 1(a,c,d) show an example of simulation results when the growth rate is *m* = 10.

Figure 1(a–d) show that SDSE, FBSE, and WSE are fundamentally consistent when describing a nearest-neighbor coupled network, a BA scale-free network, and an ER random network. In contrast, in a star network SDSE and FBSE decrease as network size increases, while the opposite is true with WSE. According to ref. 9, a star network is the most heterogeneous, thus the entropy value should decrease while the network size increases. However, we can observe from Fig. 1(b) that when WSE is applied, the entropy value of star network increases with network size. We therefore can conclude that, by using SDSE and FBSE, we can better describe star network than using WSE. It is noteworthy that after we applied standardized entropy, the network size effect is eliminate, then all three entropies (S-SDSE, S-FBSE and S-WSE) keep minimum value zero no matter how the network size changes.

As shown in Fig. 1(a–d), DDE performs different characteristics on evaluating network heterogeneity compared to the other three entropies. For a star network, WSE, SDSE, and FBSE reach the minimum value, while DDE approaches its minimum value zero as network size increases. In a nearest-neighbor coupled network, WSE, SDSE, and FBSE achieve their maximum value, while DDE always equals to zero (its minimum value). The WSE, SDSE, and FBSE of an ER random network are slightly lower but still approximately equal to their maximum value. In contrast, the DDE of an ER random network is relatively small, even smaller than DDE of a BA scale-free network with same network size and density. This phenomenon is discrepant with previous accepted view^9^.

Figure 1(e,f) show the simulation results of DDE and S-DDE of a BA scale-free network, respectively. Note that growth rate *m* ∈ [2, 18] demonstrates that changes in growth rate strongly influence DDE. Initially, DDE increases with the the network size, but when the network size reaches a certain level, the DDE has little change. In contrast, as displayed in Fig. 1(f), S-DDE decreases with the network size.

In conclusion, simulation results of typical networks demonstrate that SDSE and FBSE better reflect network heterogeneity than WSE and DDE. SDSE, FBSE, and WSE provide similar results that accurately measure the heterogeneities of the nearest-neighbor coupled network, the ER random network, and the BA scale-free network. In addition, SDSE and FBSE better describe a star network than the WSE and DDE. However, because these four well-known networks cannot discriminate between SDSE and FBSE, a new and improved benchmark network is needed.

### Simulation results of caveman network

**Figure 2:**
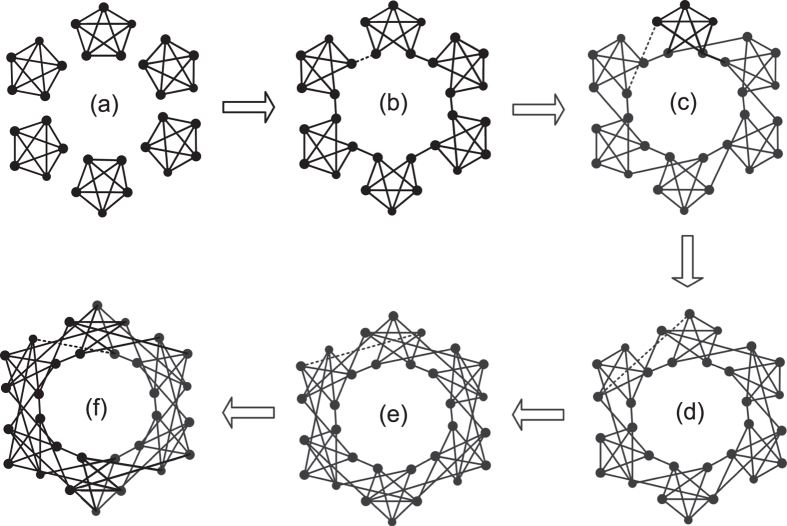
Evolution process of caveman network.

Table 1 shows the simulation results for four entropy indices using the caveman network evolutionary process shown in Figure 2. Without losing generality, we use the natural logarithm when calculating the entropy. We verify the experiments using Matlab 7.0 on a PC with an Intel^R^ Core^TM^ 2 2.40 GHz processor. When measuring DDE, the entropy value remains at 0 throughout the caveman network evolution process. Similarly, when measuring SDSE and WSE, the entropy values are unchanged ln30 during the caveman network evolution process. Thus FBSE better reflects the topological changes that occur during the evolutionary process of the caveman network, which confirms our mathematical analysis of caveman network in Methods.

**Table 1:** Structure entropy of caveman network evolutionary process.

*t*	0 (Fig. 2a)	1 (Fig. 2b)	2 (Fig. 2c)	3 (Fig. 2d)	4 (Fig. 2e)	5 (Fig. 2f)
*Entropy*						
DDE	0.00	0.00	0.00	0.00	0.00	0.00
SDSE	3.40	3.40	3.40	3.40	3.40	3.40
FBSE	3.40	**2.66**	**3.25**	3.40	3.40	3.40
WSE	3.40	3.40	3.40	3.40	3.40	3.40

**Figure 3:**
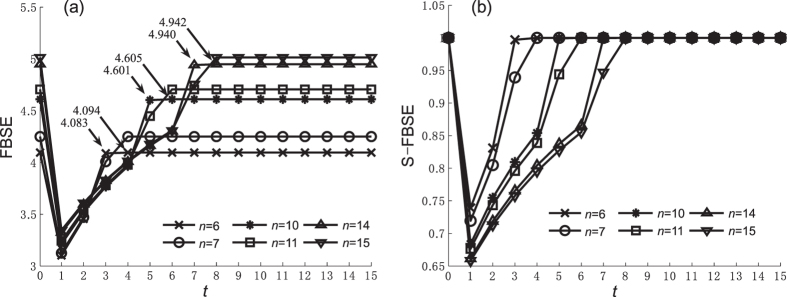
FBSE and S-FBSE of caveman network when *m* = 10. (**a**) FBFE, (**b**) S-FBFE.

**Figure 4:**
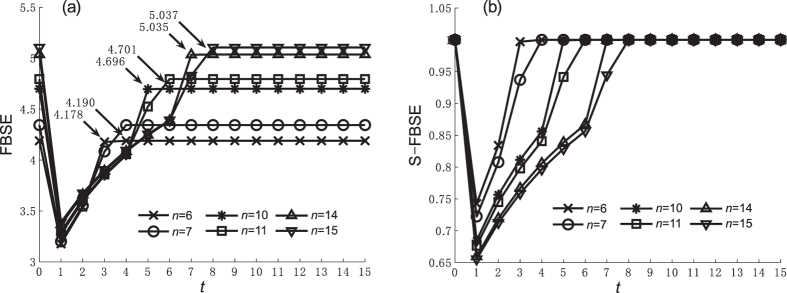
FBSE and S-FBSE of caveman network when *m* = 11. (**a**) FBFE, (**b**) S-FBFE.

**Figure 5:**
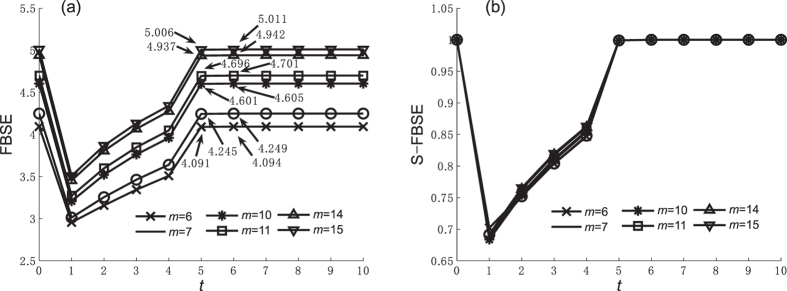
FBSE and S-FBSE of caveman network when *n* = 10. (**a**) FBFE, (**b**) S-FBFE.

**Figure 6:**
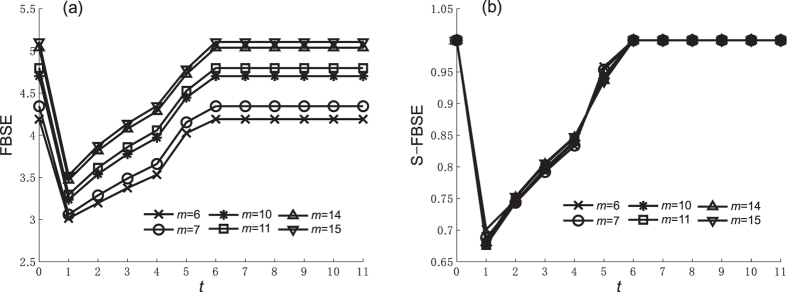
FBSE and S-FBSE of caveman network when *n* = 11. (**a**) FBFE, (**b**) S-FBFE.

We use simulations to explore how caveman network parameters affect entropy values. Because DDE, SDSE, and WSE are not affected by caveman network parameters, Figures 3, 4, 5 and 6 show the FBSE and the standardized FBSE (S-FBSE) with variable parameters. Figures 3(a) and 4(a) show the FBSE of the caveman network when *m* = 10 and *m* = 11 (*n* ∈ [5, 15] and $n\in {\mathbb{Z}}$), respectively. Note that the FBSE value at *t* = *n*/2 is close to but less than the steady state value when *n* is an even number. Note also that the FBSE value reaches log*N* when $t\ge \frac{n}{2}+1$, which agrees with our mathematical analysis of caveman network in Methods.

Figures 5(a) and 6(a) show the FBSE of a caveman network when *m* changes (*m* ∈ [5, 15] and $m\in {\mathbb{Z}}$) and *n* = 10 and *n* = 11, respectively. The caveman network reaches a steady state subject to the numerical value and the odd-even character of community node number *n*. From the FBSE of the caveman network shown in Figures 3(a), 4(a), 5(a) and 6(a), we find that during the evolutionary process entropy increases as network size *N* increases. Figures 3(b), 4(b), 5(b) and 6(b) show the respective standardized entropy values. When the standardized entropy indices are measured, the entropy value maintains a maximum value 1 when the caveman network evolution is initiated (*t* = 1), and when it reaches a steady state.

**Figure 7:**
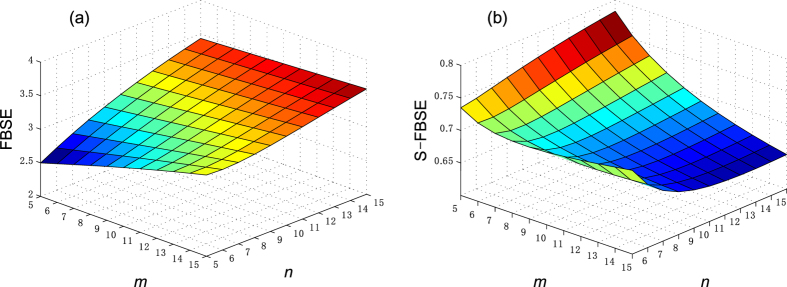
FBSE and S-FBSE of caveman network when *t* = 1. (**a**) FBFE, (**b**) S-FBFE.

Figure 7 compares the structural entropy before and after standardization at *t* = 1 when the minimum entropy value occurs. For a given community number *m* or a community node number *n*, the network size *N* increases with as *n* or *m* increases and the FBSE value then also increases. Thus the FBSE in Figure 7(a) shows network heterogeneity and also an increase in entropy under the influence of network size. If the number of communities does not change, the entropy value increases as the number of nodes in community increases. This contradicts the general understanding used in the ref. 11 analysis of the star network. Figure 7(b) shows that the standardized FBSE has some control over the entropy increase caused by network size, and thus better explains the heterogeneity of the topological structure.

## Discussion

Although entropy indices can describe some characteristics of a complex network, whether their application to network heterogeneity is accurate needs further investigation. Here we have analyzed and compared four typical static network structure entropy indices, DDE, SDSE, WSE, and FBSE. These four entropy indices were applied to four benchmark networks, the nearest-neighbor coupled network, the star network, the BA scale-free network, and the ER random network. Using mathematical analysis and simulations we found that SDSE and FBSE more accurately reflect network heterogeneity than WSE and DDE. To further distinguish local and global heterogeneity in SDSE and FBSE, we propose a caveman network and its evolutionary process rules in which the differences between network entropy indices could be found by comparing the sensitivity of indices to the evolutionary process of the network. We believe this work to be a useful exploration of the characteristics of complex networks.

## Methods

### Typical structure entropy indices

Entropy is closely related to the partition of equivalent relations in finite networks, and we use it to describe the structure and complexity of networks. An appropriately defined probability distribution, Shannon entropy is a numerical expression of network structure^18^.

Rashevsky^19^ and Trucco^20^ first used (network) entropy to measure structure complexity. Such graph variables as node number, degree sequence of node, and extended degree sequence are used to enable entropy measurement (for details about introduction of Shannon entropy, please refer to Supplementary S1). Using this information, definitions of entropy in complex networks were suggested by a consortium of scholars working in various fields. The typical entropy indices of a static network structure include the following.

#### Degree Distribution Entropy (DDE)

Under the condition that indeterminacy of the distribution probability of node number with a prescribed edge number reflects network heterogeneity, DDE is defined^10^

1 $${H}_{DD}=-\sum _{d=0}^{N-1}p(d)\mathrm{log}\,p(d),$$

where *p*(*d*) is the distribution function in which the node degree is *d* and network size is *N*. The maximum value of DDE corresponds to a network in which any two nodes have differing degree values ${H}_{DD}^{\max }=\,\mathrm{log}(N-1)$.

#### Wu Structure Entropy (WSE)

WSE characterizes network heterogeneity using the uncertainty of the distribution probability of the number of edges connected to a node and is defined^9^

2 $${H}_{Wu}=-\sum _{k=1}^{N}\frac{{d}_{k}}{{\sum }_{i=1}^{N}{d}_{i}}\,\mathrm{log}(\frac{{d}_{k}}{{\sum }_{i=1}^{N}{d}_{i}}),$$

where *d*_*k*_ is the degree value of node *k* and *N* is the network size.

#### SD Structure Entropy (SDSE)

SDSE takes both “node difference” and “edge difference” into consideration when determining network heterogeneity and is defined^11^

3 $${H}_{SD}=-\sum _{k=1}^{N}\frac{({d}_{k}+1)[1-p({d}_{k})+{\rm{\Delta }}]}{{\sum }_{i=1}^{N}\{({d}_{i}+1)[1-p({d}_{i})]+{\rm{\Delta }}\}}\,\mathrm{log}\,\frac{({d}_{k}+1)[1-p({d}_{k})+{\rm{\Delta }}]}{{\sum }_{i=1}^{N}\{({d}_{i}+1)[1-p({d}_{i})]+{\rm{\Delta }}\}},$$

where ${\rm{\Delta }} \sim O(\frac{1}{{N}^{2}})$, *N* is network size, *d*_*k*_ is the degree of node *k*, and *p*(*d*_*k*_) is the distribution probability of node degree *d*_*k*_. The minimum value of SDSE corresponds to a star network ${H}_{SD}^{\min }=\,\mathrm{log}(N+2)+\frac{2}{N+2}$ $\mathrm{log}\,\frac{N-1}{2}-\frac{N}{N+2}\,\mathrm{log}\,N$, and the maximum value corresponds to a nearest-neighbor coupled network ${H}_{SD}^{\max }=\,\mathrm{log}\,N$.

#### FB Structure Entropy (FBSE)

FBSE uses the angle of walk position^21^, combines medial and radial measurements to describe network heterogeneity, and is defined^12^

4 $${H}_{FB}=-\sum _{k=1}^{N}\frac{{\sum }_{(i,j\in S(k))}({}^{k}W_{i,j}-{}^{k}W_{i,j}^{\ast })+{d}_{k}+{\rm{\Delta }}}{{\sum }_{n=1}^{N}[{\sum }_{(i,j\in S(n))}({}^{n}W_{i,j}-{}^{n}W_{i,j}^{\ast }+{\rm{\Delta }})+{d}_{n}]}\times \,\mathrm{log}\,\frac{{\sum }_{(i,j\in S(k))}({}^{k}W_{i,j}-{}^{k}W_{i,j}^{\ast })+{d}_{k}+{\rm{\Delta }}}{{\sum }_{n=1}^{N}[{\sum }_{(i,j\in S(n))}{(}^{n}{W}_{i,j}{-}^{n}{W}_{i,j}^{\ast }+{\rm{\Delta }})+{d}_{n}]}$$

where *S*(*k*) = {(*i*, *j*) : 1 ≤ *i* ≤ *N*; 1 ≤ *j* ≤ *N*; *i* ≠ *j* ≠ *k*}, ${\rm{\Delta }} \sim O(\frac{1}{{N}^{2}})$, *N* is network size, *d*_*k*_ is the degree of node *k*, ***W*** is the maximum flow matrix, and ^*k*^ ***W*** is the matrix when line *k* and column *k* are removed from ***W***. Analogously, ^*k*^ ***W*** ^*^ represents the recalculated maximum flow matrix when line *k* and column *k* are removed from the original network.

### Structure entropy of typical networks

Network models fall into three groups. The first group is of random networks, including the random graph and its derivative models generated by statistical regularities. The second is networks with a regular structure, including the nearest-neighbor coupled network, the fully connected network, and the star network. The third is networks that combine some features from random and regular networks, such as scale-free networks and small-world networks.

Here we examine the structure entropy of four typical networks, the ER random network, the nearest-neighbor coupled network, the star network, and the BA scale-free network.

#### Mathematical Analysis of Typical Networks

- Structure Entropy of ER Random Network.
  The ER random network is an important reference model introduced by Erdos and Renyi in 1906^13^. The model supplies an equal probability network ($$\begin{array}{c}N(N-1)/2\\ n\end{array}$$) with an average degree 〈*d*〉 = *q*(*N* − 1) and a degree distribution that is a Poisson distribution: *P*(*d*) ≈ *e* ^−〈*d*〉^ 〈*d*〉^*d*^/*d*!. Here *N* is the network size and *n* stands for the total number of edges. Thus the SDSE of an ER random network is
  5 $$\begin{array}{rcl}{H}_{SD} & = & -\sum _{i=0}^{N-1}N\cdot ({e}^{-\langle d\rangle }{\langle d\rangle }^{i}/i!)\cdot \{\frac{(i+1)(1-{e}^{-\langle d\rangle }{\langle d\rangle }^{i}/i!+{\rm{\Delta }})}{{\sum }_{d\mathrm{=0}}^{N-1}[N\cdot (d+1)\cdot ({e}^{-\langle d\rangle }{\langle d\rangle }^{d}/d!)\cdot (1-{e}^{-\langle d\rangle }{\langle d\rangle }^{d}/d!+{\rm{\Delta }})]}\}\\  &  & \,\cdot \{\mathrm{log}\,\frac{(i+1)(1-{e}^{-\langle d\rangle }{\langle d\rangle }^{i}/i!+{\rm{\Delta }})}{{\sum }_{d=0}^{N-1}[N\cdot (d+1)\cdot ({e}^{-\langle d\rangle }{\langle d\rangle }^{d}/d!)\cdot (1-{e}^{-\langle d\rangle }{\langle d\rangle }^{d}/d!+{\rm{\Delta }})]}\}\end{array},$$
  the FBSE of an ER random network is
  6 $$\begin{array}{ccc}{H}_{FB} & = & -\sum _{k=1}^{N}\frac{{{e}_{N-1}}^{{\rm{T}}}\cdot {}_{}^{k}{\boldsymbol{W}}\cdot {e}_{N-1}-{e}_{N-1}\cdot {}_{}^{k}{{\boldsymbol{W}}}^{\ast }\cdot {e}_{N-1}+2{{e}_{N}}^{{\rm{T}}}\cdot {\boldsymbol{A}}{\delta }_{k}+{\rm{\Delta }}}{2N\langle k\rangle +\sum _{n=1}^{N}({{e}_{N-1}}^{{\rm{T}}}\cdot {}_{}^{n}{\boldsymbol{W}}\cdot {e}_{N-1}-{e}_{N-1}\cdot {}_{}^{n}{{\boldsymbol{W}}}^{\ast }\cdot {e}_{N-1}+{\rm{\Delta }})}\\  &  & \times {\rm{l}}{\rm{o}}{\rm{g}}\frac{{{e}_{N-1}}^{{\rm{T}}}\cdot {}_{}^{k}{\boldsymbol{W}}\cdot {e}_{N-1}-{e}_{N-1}\cdot {}_{}^{k}{{\boldsymbol{W}}}^{\ast }\cdot {e}_{N-1}+2{{e}_{N}}^{{\rm{T}}}\cdot {\boldsymbol{A}}{\delta }_{k}+{\rm{\Delta }}}{2N\langle k\rangle +\sum _{n=1}^{N}({{e}_{N-1}}^{{\rm{T}}}\cdot {}_{}^{n}{\boldsymbol{W}}\cdot {e}_{N-1}-{e}_{N-1}\cdot {}_{}^{n}{{\boldsymbol{W}}}^{\ast }\cdot {e}_{N-1}+{\rm{\Delta }})}\end{array},$$
  the DDE of an ER random network is
  7 $${H}_{DD}=\sum _{d=0}^{N-1}({e}^{-\langle d\rangle }{\langle d\rangle }^{d}/d!)\,{\rm{l}}{\rm{o}}{\rm{g}}({e}^{-\langle d\rangle }{\langle d\rangle }^{d}/d!),$$
  and the WSE of an ER random network is
  8 $$\begin{array}{rcl}{H}_{Wu} & = & -\sum _{d=0}^{N-1}N\cdot P(d)\cdot \frac{d}{N\cdot \langle d\rangle }\,\mathrm{log}[N\cdot P(d)\cdot \frac{d}{N\cdot \langle d\rangle }]\\  & = & \sum _{d=0}^{N-1}\frac{{e}^{-\langle d\rangle }{\langle d\rangle }^{d-1}}{(d-1)!}\mathrm{log}[\frac{{e}^{-\langle d\rangle }{\langle d\rangle }^{d-1}}{(d-1)!}]\end{array}.$$
- Structure Entropy of Regular Network.
  A nearest-neighbor coupled network is a typical regular network in which each node only connects with its neighbor node. For a given *k* (that is an even number), all nodes in the network form a circle in which each node only connects on two sides with its *k*/2 neighbor nodes. When *k* is sufficiently large, it has a high cluster index *C* = 3(*k* − 2)/4(*k* − 1) ≈ 0.75, and when *N* is sufficiently large, it has a large average path length *L* ≈ *N*/2*k*, where *N* is the network size.
  SDSE, FBSE, and WSE all satisfy inequality *H* ≤ log*N*. For SDSE, *H* = log*N* only when (*d*_*k*_ + 1)[1 − *p*(*d*_*k*_) + Δ] remains the same value for any given *k* ∈ {1, 2, …, *N*}. Similarly, for FBSE, ${{e}_{N-1}}^{{\rm{T}}}\cdot {}_{}^{k}W\cdot {e}_{N-1}-{e}_{N-1}\cdot {}_{}^{k}{W}^{\ast }\cdot {e}_{N-1}\,+$ ${{e}_{N}}^{{\rm{T}}}A{\delta }_{k}+{{e}_{N}}^{{\rm{T}}}{A}^{{\rm{T}}}{\delta }_{k}$ stays the same, corresponding to any *k* ∈ {1, 2, …, *N*}, then *H* = log*N*. For WSE, *d*_*k*_ is unchanged for any *k* ∈ {1, 2, …, *N*}, and *H* = log*N*. For a given network size *N*, all nodes in a nearest-neighbor coupled network are structurally equivalent, so
  9 $${H}_{SD}^{\max }={H}_{FB}^{\max }={H}_{Wu}^{\max }=-\sum _{i=1}^{N}\frac{1}{N}\mathrm{log}\,\frac{1}{N}=\,\mathrm{log}\,N.$$
  Unlike these listed entropies, the minimum value of DDE corresponds to the nearest-neighbor coupled network: ${H}_{DD}^{{\rm{\min }}}=0$.
  In a star network all nodes are connected to one central node and have no other connections, i.e., to each other. If for a given network size *N* the degree of the central node is *d*_1_, then *d*_1_ = *N* − 1, *d*_*i*_ = 1(*i* ≠ 1). In both FBSE and SDSE the minimum value of the network entropy is in the star network, and both share the same analytic expression,
  10 $$\begin{array}{ccc}{H}_{SD}^{min} & = & {H}_{FB}^{min}=-[\frac{N}{N+2}\,{\rm{l}}{\rm{o}}{\rm{g}}\,\frac{N}{N+2}+(N-1)\\  &  & \times \frac{2}{(N-1)(N+2)}\,{\rm{l}}{\rm{o}}{\rm{g}}\,\frac{2}{(N-1)(N+2)}]\\  & = & {\rm{l}}{\rm{o}}{\rm{g}}(N+2)+\frac{2}{N+2}\,{\rm{l}}{\rm{o}}{\rm{g}}\,\frac{N-1}{2}-\frac{N}{N+2}\,{\rm{l}}{\rm{o}}{\rm{g}}\,N\end{array}.$$
  The minimum value of WSE also corresponds to a star network: ${H}_{Wu}^{\min }=\frac{\mathrm{log}\,4(n-1)}{2}$, and the DDE of a star network is ${H}_{DD}=\,\mathrm{log}\,N-\frac{N-1}{N}\,\mathrm{log}(N-1)$.
- Structure Entropy of BA Scale-Free Network.

The BA scale-free network^14^ was proposed by Barabasi and Albert in 1999. In a BA scale-free network the initial node is *m*_0_ and the growth rate is *m*. After a time *t*, the network transforms into a scale-free network of *N* = *t* + *m*_0_ nodes and *mt* edges with a degree distribution $p(d) \sim 2{m}^{2}{d}^{-3}$. Unlike the Poisson distribution of a random network, a scale-free network with a power-law distribution has an inhomogeneous structure. In a scale-free network, the function of the degree distribution has no peak value. Most nodes have few connections and a small number have many connections.

The SDSE of a BA scale-free network at time *t* can be expressed

11 $${H}_{SD}(t)=-\sum _{k=1}^{N}\frac{m{(\frac{t}{{t}_{k}})}^{\frac{1}{2}}+1-2{m}^{-1}{(\frac{t}{{t}_{k}})}^{-\frac{3}{2}}-2{(\frac{t}{{t}_{k}})}^{-1}}{2mt-\frac{4}{5}{m}^{-1}t}\,\mathrm{log}\,\frac{m{(\frac{t}{{t}_{k}})}^{\frac{1}{2}}+1-2{m}^{-1}{(\frac{t}{{t}_{k}})}^{-\frac{3}{2}}-2{(\frac{t}{{t}_{k}})}^{-1}}{2mt-\frac{4}{5}{m}^{-1}t}$$

where *t*_*k*_ is the time when node *k* joins the network. And the FBSE of a BA scale-free network at time *t* can be expressed

12 $$\begin{array}{ccc}{H}_{FB}(t) & = & -\sum _{k=1}^{{m}_{0}+t}\frac{{{{\boldsymbol{e}}}_{{m}_{0}+t-1}}^{{\rm{T}}}\cdot {}_{t}^{k}{\boldsymbol{W}}\cdot {{\boldsymbol{e}}}_{{m}_{0}+t-1}-{{\boldsymbol{e}}}_{{m}_{0}+t-1}\cdot {}_{t}^{k}{{\boldsymbol{W}}}^{\ast }\cdot {{\boldsymbol{e}}}_{{m}_{0}+t-1}+2{{{\boldsymbol{e}}}_{{m}_{0}+t}}^{{\rm{T}}}\cdot {\boldsymbol{A}}(t)\cdot {\delta }_{k}+{\rm{\Delta }}}{4mt+\sum _{n=1}^{{m}_{0}+t}({{{\boldsymbol{e}}}_{{m}_{0}+t-1}}^{{\rm{T}}}\cdot {}_{t}^{n}{\boldsymbol{W}}\cdot {{\boldsymbol{e}}}_{{m}_{0}+t-1}-{{\boldsymbol{e}}}_{{m}_{0}+t-1}\cdot {}_{t}^{n}{{\boldsymbol{W}}}^{\ast }\cdot {{\boldsymbol{e}}}_{{m}_{0}+t-1}+{\rm{\Delta }})}\\  &  & \times \,{\rm{l}}{\rm{o}}{\rm{g}}\,\frac{{{{\boldsymbol{e}}}_{{m}_{0}+t-1}}^{{\rm{T}}}\cdot {}_{t}^{k}{\boldsymbol{W}}\cdot {{\boldsymbol{e}}}_{{m}_{0}+t-1}-{{\boldsymbol{e}}}_{{m}_{0}+t-1}\cdot {}_{t}^{k}{{\boldsymbol{W}}}^{\ast }\cdot {{\boldsymbol{e}}}_{{m}_{0}+t-1}+2{{{\boldsymbol{e}}}_{{m}_{0}+t}}^{{\rm{T}}}\cdot {\boldsymbol{A}}(t)\cdot {\delta }_{k}+{\rm{\Delta }}}{4mt+\sum _{n=1}^{{m}_{0}+t}({{{\boldsymbol{e}}}_{{m}_{0}+t-1}}^{{\rm{T}}}\cdot {}_{t}^{n}{\boldsymbol{W}}\cdot {{\boldsymbol{e}}}_{{m}_{0}+t-1}-{{\boldsymbol{e}}}_{{m}_{0}+t-1}\cdot {}_{t}^{n}{{\boldsymbol{W}}}^{\ast }\cdot {{\boldsymbol{e}}}_{{m}_{0}+t-1}+{\rm{\Delta }})}\end{array},$$

where ***e***_*N*_ is an *N*-dimensional column vector in which all elements equal 1, and ${{{\boldsymbol{e}}}_{N}}^{T}$ is the transposed vector of *e*_*N*_. ***A*** is the adjacency matrix of network *D*, and ***A*** ^*T*^ is the transposed matrix of ***A***. *δ*_*k*_ is a *N*-dimensional column vector in which element *k* is 1 and others are 0.

Similarly, the WSE of a BA scale-free network at time *t* is

13 $${H}_{Wu}(t)=-\sum _{k=1}^{N}\frac{m{(\frac{t}{{t}_{k}})}^{1/2}}{2mt}\,\mathrm{log}\,\frac{m{(\frac{t}{{t}_{k}})}^{1/2}}{2mt}.$$

At time *t*, the DDE of a BA scale-free network is

14 $${H}_{DD}=-\sum _{d=0}^{N-1}2{m}^{2}{d}^{-3}\,\mathrm{log}\,2{m}^{2}{d}^{-3}.$$

### Structure entropy of the caveman network

Simulation results on the four classical networks discussed above demonstrate that SDSE and FBSE can describe the heterogeneity of various networks, but to further probe these two entropy indices we construct here the evolutionary process provided by a caveman network. In an evolutionary caveman network the overall topological structure of the network changes, but such local features as node degree value and network degree distribution do not.

#### Construction and Evolution of Caveman Network

A caveman network is a regular network that serves as a benchmark to reflect community structure and has a high local clustering coefficient and a long average path length^16, 22^. A caveman network is divided into several centrally connected sub-networks with few connections to each other. In the traditional caveman network, node degree is not restricted and thus can vary. Thus as in the benchmark networks discussed above, in a caveman network it is difficult to determine whether the local node degree or the global connection mode contributes to structural heterogeneity. In addition, because the structure is fixed, no evolution occurs, and comparing entropy indices is difficult in a traditional caveman network, we must redefine it.

To test the sensitivity of different entropy values to network evolution, we examine the continuous evolution rules of a caveman network. Network size *N* and number of community nodes *n* conform to *N*|*n*. Each node *k* is *i* ~ *j*. Nodes with the same *i* value are designated a community in which $i=\lceil \frac{k}{n}\rceil$ ($\lceil \,\rceil$ means rounding up to an integer), *j* = *k* − (*i* − 1)*n, 0 < *i* ≤ *m*, 0 < *j* ≤ *n*, *m* ≥ 5, *n* ≥ 4 and $i,j\in {\mathbb{Z}}$. An edge between node ${i}_{1} \sim {j}_{1}$ and node ${i}_{2} \sim {j}_{2}$ is designated an intra-community edge when *i*_1_ = *i*_2_ or an inter-community edge when *i*_1_ ≠ *i*_2_. The *t* value is the evolution time and the termination time is *T* ≤ *n*. There several steps in the caveman evolution process:

**Step 1:** When *t* = 0, the network is an m fully-connected n-regular network. When ∀*i* ∈ [0, *m*], $i\in {\mathbb{Z}}$, $i \sim {\tau }_{1}$ and $i \sim {\tau }_{2}$ are connected, where 0 < *τ*_1_, *τ*_2_ ≤ *n*, *τ*_1_ ≠ *τ*_2_, ${\tau }_{1},\,{\tau }_{2}\in {\mathbb{Z}}$.

**Step 2:** When 1 ≤ *t* ≤ *n* − 1, the intra-community edges gradually become inter-community edges. When ∀*τ* ∈ {*i*}, $\tau  \sim t$ and $\tau  \sim (t+1)$ are disconnected. When ∀*τ* ∈ {*i*} − {*m*}, $\tau  \sim (t+1)$ and $(\tau +1) \sim t$ are connected and $m \sim (t+1)$ and $1 \sim t$ are connected.

**Step 3:** When *t* = *T* = *n*, when ∀*τ* ∈ {*i*}, $\tau  \sim t$ and $\tau  \sim 1$ are disconnected. When ∀*τ* ∈ {*i*} − {*m*}, $\tau  \sim 1$ and $(\tau +1) \sim t$ are connected and $m \sim 1$ and $1 \sim t$ are connected.

Figure 2 shows the evolutionary process of a caveman network of network size *N* = 30. There are six communities and each community has five nodes. Figure 2(a) is the initial network at *t* = 0 that consists of six independent fully-connected networks of size *n* = 5. All the nodes in Figure 2(a) have the same degree value and are structurally equivalent. Figure 2(b) is the caveman network at *t* = 1 during which one intra-community edge of each community disconnects and connects with a neighbor community. Although every node still has the same degree value, the topological structure of the network has changed, and the nodes with inter-community edges are better able to communicate than those without inter-community edges. As *t* increases, the number of nodes with inter-community edges also increases. Figure 2(f) shows that when *t* = 5 the evolutionary process concludes, all nodes have two inter-community edges and two intra-community edges, and the caveman network has regained equilibrium.

At time *t* in the caveman network evolution, ${\theta }_{{k}_{out},t}$ is the number of nodes with *k*_*out*_ inter-community edges in a given community, and ${\theta }_{k,out}^{t}$ is the number of inter-community edges of node *k*. When *k*_*out*_ = 0,

15 $${\theta }_{{k}_{out},t}=\{\begin{array}{cc}n, & t=0\\ n-(t+1), & t\in \{1,2,\cdot \cdot \cdot ,n-1\}\\ 0, & t=n\end{array},$$

When *k*_*out*_ = 1,

16 $${\theta }_{{k}_{out},t}=\{\begin{array}{cc}0, & t\in \{0,n\}\\ 2, & t\in \{1,2,\cdot \cdot \cdot ,n-1\}\end{array},$$

When *k*_*out*_ = 2,

17 $${\theta }_{{k}_{out},t}=\{\begin{array}{cc}0, & t=0\\ t-1, & t\in \{1,2,\cdot \cdot \cdot ,n-1\}\\ n, & t=n\end{array}.$$

#### Mathematical Analysis of Caveman Network

To test the sensitivity of different entropy indices to changes in the network topological structure, we examine the entropy value changes during in the network evolution process. In a caveman network with a community number *m* and node number *n* (the network size is *N* = *m* * *n*), *H*_*DD*_(*t*), *H*_*SD*_(*t*), *H*_*Wu*_(*t*), and *H*_*FB*_(*t*) are the entropy values of DDE, SDSE, WSE, and FBSE at time *t*, respectively. Because node degree value and degree distribution do not change with time *t* in a caveman network, we conclude that *H*_*DD*_(*t*) ≡ 0, *H*_*SD*_(*t*) = *H*_*Wu*_(*t*) ≡ log*N*.

Using FBSE, a caveman network has *m* fully-connected sub-networks, every node is equal at *t* = 0, and there is no heterogeneity. Thus *H*_*FB*_(0) = log*N*.

When *t* = 1 the topological structure changes and dominant nodes with an inter-community edge appear in every community. These nodes are more able to control network flow than nodes with no inter-community edges, and thus the network is highly heterogeneous. As evolution continues, the number of nodes with inter-community edges increases, the control privilege of network flow evens out, and the heterogeneity weakens. When $t\ge \frac{n+1}{2}$, the caveman network regains its non-heterogeneity. The FBSE of the caveman network in steady state (*n*/2 < *t* ≤ *n*) is *H*_*FB*_(*t*) = log*N*. For more details about FB structure entropy solution, see Supplementary S2; for FB structure entropy of caveman network in non-steady states process, refer to Supplementary S3; and for FB structure entropy of caveman network in steady states process, refer to Supplementary S4.

## Electronic supplementary material

Supplementary Information
