# Supplementary material for: Analysis and evaluation of the entropy indices of a static network structure
Source: Sci Rep. 2017 Aug 24;7:9340. doi: 10.1038/s41598-017-09475-9 (PMC5570930; doi:10.1038/s41598-017-09475-9)
Supplement: Supplementary file 1 — Supplementary Information [file 41598_2017_9475_MOESM1_ESM.pdf]

Analysis and evaluation of the entropy indices of a static network structure

Meng Cai [1, 3*+], Ying Cui[2, 3*+], H. Eugene Stanley[3]

[1]School of Economics and Management, Xidian University, Xi'an 710071, China;

[2]School of Mechano-Electronic Engineering, Xidian University, Xi'an 710071, China;

[3]Center for Polymer Studies and Department of Physics, Boston University, Boston, Massachusetts 02215, USA.)

*corresponding authors: mcai@xidian.edu.cn (M.C.), cuiyingauto@gmail.com (Y.C.)

[+]these authors contributed equally to this work

Supplementary Information

S1. Introduction of Shannon entropy

An appropriately defined probability distribution, Shannon entropy is a numerical expression of network structure[1]. Here X is the invariant of a network, α divides X into k subsets, and $|X_i|$ is the potential of subset i. Entropy can be defined[2]

$$H(\mathrm{G},\alpha) = |X|\log(|X|) - \sum_{i=1}^{k}|X_i|\log(|X_i|),$$

$$\text{and } \bar{H}(\mathrm{G},\alpha) = -\sum_{i=1}^{k} P_i \log(P_i) = -\sum_{i=1}^{k}\frac{|X_i|}{|X|}\log\left(\frac{|X_i|}{|X|}\right),$$

where $G = (V,E), |V| < \infty, E \subseteq \binom{V}{2}$ is a finite undirected graph and $\mathrm{G} = (V,E), |V| < \infty, E \subseteq V \times V$ is a finite directed graph.

Rashevsky[3] and Trucco[4] first used (network) entropy to measure structure complexity. Such graph variables as node number, degree sequence of node, and extended degree sequence are used to enable entropy measurement. These measurements were first introduced by Rashevsky to provide topological information on graph G, defined as[3]

$${}^{V}H(\mathrm{G}) := |V|\log(|V|) - \sum_{i=1}^{k}|N_i|\log(|N_i|),$$

$$\text{and } {}^{V}\bar{H}(\mathrm{G}) := -\sum_{i=1}^{k}\frac{|N_i|}{|V|}\log\left(\frac{|N_i|}{|V|}\right).$$

[1] da Silva, V. d. P. R., Belo Filho, A. F., Almeida, R. S. R., de Holanda, R. M. & da Cunha Campos, J. H. B. Shannon information entropy for assessing space–time variability of rainfall and streamflow in semiarid region. *Sci. The Total. Environ.* **544**, 330–338 (2016).

[2] Sandhu, R. et al. Graph curvature for differentiating cancer networks. *Sci. reports* **5**, 12323 (2015).

[3] Rashevsky, N. Life, information theory, and topology. *Bull. Math. Biol.* **17**, 229–235 (1955).

[4] Trucco, E. A note on the information content of graphs. *Bull. Math. Biol.* **18**, 129–135 (1956).

S2. Partial evaluation results of FBSE in caveman network

For a caveman network with network size *N* and community number *n*, to any node *k* denoted by $\tau_1 \sim \tau_2$, it has

$$b_k = \sum_{(i,j \in S(k))} \left({}^{k}\boldsymbol{W}_{i,j} - {}^{k}\boldsymbol{W}^{*}_{i,j} \right)$$

$$S(k) = \{(i,j): 1 \le i \le N; 1 \le j \le N; i \ne j \ne k\}$$

Where b_k (called flow betweenness) means the variation of maximum flow when network node *k* is removed from the network. $\boldsymbol{W}$ is the maximum flow matrix and ${}^{k}\boldsymbol{W}$ means the matrix by removing the *k*th line and *k*th column from $\boldsymbol{W}$. Analogously, ${}^{k}\boldsymbol{W}^{*}$ represents the recalculated maximum flow matrix after the removal of the *k*th line and *k*th column from the original network.

Based on the symmetry of network, it is not difficult to understand the fundamental theorem hereinafter illustrated:

Theorem 1: for $\forall i_1, \forall i_2,\ 0 < i_1, i_2 \le m$, then $b_{i_1 \sim j} = b_{i_2 \sim j}, 0 < j \le n$.

Theorem 2: for $\forall k_1, \forall k_2,\ 0 < k_1, k_2 \le N$, if $\theta^{t}_{k_1,\mathrm{out}} = \theta^{t}_{k_2,\mathrm{out}}$, then $b_{k_1} = b_{k_2}$.

Theorem 3: for $\forall i, \forall j, \forall k,\ 0 < i, j, k \le N, i \ne j \ne k$, then $m_k(i,j) = m_k(j,i)$.

Where $m_k(i,j)$ is the flow quantity when the maximum flow passes node *k* from node *i* to node *j*. $\theta^{t}_{k,\mathrm{out}}$ is denoted as the number of inter-community edges of node *k*.

Let $S(k) = \bigcup_{\varphi=1}^{4} S_i(k)$, where

$$S_1(k) = \left\{(i,j): \left\lceil \frac{i}{n} \right\rceil = \tau_1; \left\lceil \frac{j}{n} \right\rceil = \tau_1 \right\} \bigcap S(k)$$

$$S_2(k) = \left\{(i,j): \left\lceil \frac{i}{n} \right\rceil = \tau_1; \left\lceil \frac{j}{n} \right\rceil \ne \tau_1 \right\} \bigcap S(k)$$

$$S_3(k) = \left\{(i,j): \left\lceil \frac{i}{n} \right\rceil \ne \left\lceil \frac{j}{n} \right\rceil \ne \tau_1 \right\} \bigcap S(k)$$

$$S_4(k) = \left\{(i,j): \left\lceil \frac{i}{n} \right\rceil = \left\lceil \frac{j}{n} \right\rceil \ne \tau_1 \right\} \bigcap S(k)$$

It is not difficult to find that $\bigcap_{\varphi=1}^{4} S_i(k) = \emptyset$, Thus,

$$b_k = \Psi_1 + \Psi_2 + \Psi_3 + \Psi_4$$

I. When $t = 0$, for $\forall k \in \{i \sim j | k_{\text{out}} = 0\}$, it has

$$\Psi_1 = \mathrm{A}_{n-1}^2$$

$$\Psi_2 = \Psi_3 = \Psi_4 = 0$$

II. When $t = 1$, for $\forall k \in \{i \sim j | k_{\text{out}} = 0\}$, it has

$$\Psi_1 = \mathrm{A}_{n-1}^2$$

$$\Psi_2 = \Psi_3 = \Psi_4 = 0$$

When $t = 1$, for $\forall k \in \{i \sim j | k_{\text{out}} = 1\}$, it has

$$\Psi_1 = \mathrm{A}_{n-1}^2$$

$$\Psi_2 = 2(m-1) \cdot \mathrm{C}_{n-1}^1 \cdot \mathrm{C}_n^1$$

$$\Psi_3 = \mathrm{A}_{\mathrm{m}-1}^2 \cdot \mathrm{C}_{\mathrm{n}}^1 \cdot \mathrm{C}_{\mathrm{n}}^1$$

$$\Psi_4 = (m-1) \cdot (2\mathrm{C}_{n-2}^1 + 2\mathrm{C}_{n-2}^1 + \mathrm{A}_2^2)$$

III. When $t = 2$, for $\forall k \in \{i \sim j | k_{\text{out}} = 0\}$, it has

$$\Psi_1 = \mathrm{A}_{n-1}^2$$

$$\Psi_2 = \Psi_3 = \Psi_4 = 0$$

When $t = 2$, for $\forall k \in \{i \sim j | k_{\text{out}} = 1\}$, it has

$$\Psi_1 = \mathrm{A}_{n-1}^2$$

$$\Psi_2 = 2(m-1) \cdot \mathrm{C}_{n-1}^1 \cdot \mathrm{C}_n^1$$

$$\Psi_3 = \mathrm{A}_{m-1}^2 \cdot \mathrm{C}_n^1 \cdot \mathrm{C}_n^1$$

$$\Psi_4 = 2\mathrm{C}_{n-1}^1$$

When $t = 2$, for $\forall k \in \{i \sim j | k_{\text{out}} = 2\}$, it has

$$\Psi_1 = \mathrm{A}_{n-1}^2 - 2\theta_{k_{\text{out}},t}$$

$$\Psi_2 = 2k_{\text{out}}(m-1) \cdot \mathrm{C}_{n-1}^1 \cdot \mathrm{C}_n^1$$

$$\Psi_3 = \mathrm{A}_{m-1}^2 \cdot \mathrm{C}_n^1 \cdot \mathrm{C}_n^1$$

$$\Psi_4 = 2\theta_{k_{\text{out}}-1,\mathrm{t}}(\mathrm{A}_2^2 \cdot \mathrm{C}_{n-1}^1 - 1)$$

IV. When $3 \le t \le \frac{n}{2} - 1$, for $\forall k \in \{i \sim j | k_{\text{out}} = 0\}$, it has

$$\Psi_1 = \mathrm{A}_{n-1}^2$$

$$\Psi_2 = \Psi_3 = \Psi_4 = 0$$

When $3 \le t \le \frac{n}{2} - 1$, for $\forall k \in \{i \sim j | k_{\text{out}} = 1\}$, it has

$$\Psi_1 = \mathrm{A}_{n-1}^2$$

$$\Psi_2 = 2(m-1) \cdot \mathrm{C}_{n-1}^1 \cdot \mathrm{C}_n^1$$

$$\Psi_3 = \mathrm{A}_{m-1}^2 \cdot \mathrm{C}_n^1 \cdot \mathrm{C}_n^1$$

$$\Psi_4 = 2\mathrm{C}_{n-1}^1$$

When $3 \le t \le \frac{n}{2} - 1$, for $\forall k \in \{i \sim j | k_{\text{out}} = 2\}$, it has

$$\Psi_1 = \mathrm{A}_{n-1}^2 - 2$$

$$\Psi_2 = 2k_{\text{out}}(m-1) \cdot \mathrm{C}_{n-1}^1 \cdot \mathrm{C}_n^1$$

$$\Psi_3 = \mathrm{A}_{m-1}^2 \cdot \mathrm{C}_n^1 \cdot \mathrm{C}_n^1$$

$$\Psi_4 = 2\theta_{k_{\text{out}}-1,t}\mathrm{C}_{n-1}^1$$

V. When $\frac{n}{2} - 1 < t \le \frac{n}{2}$, for $\forall k \in \{i \sim j | k_{\text{out}} = 0\}$, it has

i. When *n* is an odd number, which means $t = \frac{n-1}{2}$

$$\Psi_1 = \mathrm{A}_{n-1}^2$$

$$\Psi_2 = 2(m-1) \cdot \mathrm{C}_{n-1}^1 \cdot \mathrm{C}_n^1$$

$$\Psi_3 = \Psi_4 = 0$$

ii. When *n* is an even number, which means $t = \frac{n}{2}$

$$\Psi_1 = \mathrm{A}_{n-1}^2$$

$$\Psi_2 = 2(m-1) \cdot \mathrm{C}_{n-1}^1 \cdot \mathrm{C}_n^1$$

$$\Psi_3 = \Psi_4 = 0$$

When $\frac{n}{2} - 1 < t \le \frac{n}{2}$, for $\forall k \in \{i \sim j | k_{\text{out}} = 1\}$, it has

i. When *n* is an odd number, which means $t = \frac{n-1}{2}$

$$\Psi_1 = \mathrm{A}_{n-1}^2$$

$$\Psi_2 = 2(m-1) \cdot \mathrm{C}_{n-1}^1 \cdot \mathrm{C}_n^1$$

$$\Psi_3 = \mathrm{A}_{m-1}^2 \cdot \mathrm{C}_n^1 \cdot \mathrm{C}_n^1$$

$$\Psi_4 = 2\mathrm{C}_{n-1}^1$$

ii. When *n* is an even number, which means $t = \frac{n}{2}$

$$\Psi_1 = \mathrm{A}_{n-1}^2$$

$$\Psi_2 = 2[(m-1)\cdot \mathrm{C}_{n-2}^1 \cdot \mathrm{C}_n^1 + k_{\mathrm{out}}]$$

$$\Psi_3 = 2k_{\mathrm{out}}\mathrm{C}_{m-2}^1 \cdot \mathrm{C}_n^1$$

$$\Psi_4 = 2k_{\mathrm{out}}\mathrm{C}_{n-1}^1$$

When $\frac{n}{2}-1 < t \leq \frac{n}{2}$, for $\forall k \in \{i\sim j | k_{\mathrm{out}} = 2\}$, it has

i. When *n* is an odd number, which means $t = \frac{n-1}{2}$

$$\Psi_1 = \mathrm{A}_{n-1}^2 - 2$$

$$\Psi_2 = 2k_{\mathrm{out}}(m-1)\cdot \mathrm{C}_{n-1}^1 \cdot \mathrm{C}_n^1$$

$$\Psi_3 = \mathrm{A}_{m-1}^2 \cdot \mathrm{C}_n^1 \cdot \mathrm{C}_n^1$$

$$\Psi_4 = 2\theta_{k_{\mathrm{out}}-1,t}\mathrm{C}_{n-1}^1$$

ii. When *n* is an even number, which means $t = \frac{n}{2}$

$$\Psi_1 = \mathrm{A}_{n-1}^2 - 2$$

$$\Psi_2 = 2(m-1)\cdot \mathrm{C}_{n-1}^1 \cdot \mathrm{C}_n^1$$

$$\Psi_3 = 2[k_{\mathrm{out}}\mathrm{C}_{m-2}^1 \cdot \mathrm{C}_n^1 - 1]$$

$$\Psi_4 = 2k_{\mathrm{out}}\mathrm{C}_{n-1}^1$$

VI. When $\frac{n}{2} < t \leq n-2$, for $\forall k \in \{i\sim j | k_{\mathrm{out}} = 0\}$, it has

$$\Psi_1 = \mathrm{A}_{n-1}^2$$

$$\Psi_2 = 2(m-1)\cdot \mathrm{C}_{n-1}^1 \cdot \mathrm{C}_n^1$$

$$\Psi_3 = \Psi_4 = 0$$

When $\frac{n}{2} < t \leq n-1$, for $\forall k \in \{i\sim j | k_{\mathrm{out}} = 1\}$, it has

$$\Psi_1 = \mathrm{A}_{n-1}^2$$

$$\Psi_2 = 2[(m-1)\cdot \mathrm{C}_{n-2}^1 \cdot \mathrm{C}_n^1 + k_{\mathrm{out}}]$$

$$\Psi_3 = 2k_{\mathrm{out}}\mathrm{C}_{m-2}^1 \cdot \mathrm{C}_n^1$$

$$\Psi_4 = 2k_{\mathrm{out}}\mathrm{C}_{n-1}^1$$

When $\frac{n}{2} < t \leq n$, for $\forall k \in \{i\sim j | k_{\mathrm{out}} = 2\}$, it has

$$\Psi_1 = \mathrm{A}_{n-1}^2 - 2$$

$$\Psi_2 = 2\left[(m-1)\cdot \mathrm{C}_{n-1-k_{\mathrm{out}}}^1 \cdot \mathrm{C}_n^1 + 2k_{\mathrm{out}}\right]$$

$$\Psi_3 = 2[k_{\mathrm{out}}\mathrm{C}_{m-2}^1 \cdot \mathrm{C}_n^1 - 1]$$

$$\Psi_4 = 2k_{\text{out}}\text{C}_{n-1}^1$$

S3. FBSE of caveman network in non-steady states

Based on partial evaluation results of FBSE discussed in S1, FBSE of caveman network in non-steady states can be calculated by

$$\begin{aligned}
&H_{\text{FB}}(1) \\
&= -m\left\{\frac{2[(m^2-m+1)n^2+2(m-2)n+7-6m]}{2m[(m^2-m+1)n^2+(2m-5)n+8-6m]+m(n-1)(n-2)^2+(n-1)N}\right. \\
&\times \log\frac{(m^2-m+1)n^2+2(m-2)n+7-6m}{2m[(m^2-m+1)n^2+(2m-5)n+8-6m]+m(n-1)(n-2)^2+(n-1)N} \\
&+ \frac{(n-1)^2(n-2)}{2m[(m^2-m+1)n^2+(2m-5)n+8-6m]+m(n-1)(n-2)^2+(n-1)N} \\
&\left.\times \log\frac{(n-1)^2}{2m[(m^2-m+1)n^2+(2m-5)n+8-6m]+m(n-1)(n-2)^2+(n-1)N}\right\}
\end{aligned}$$

$$\begin{aligned}
&H_{\text{FB}}(2) \\
&= -m\left\{\frac{(m^2+m-1)n^2+2(5-2m)n-13}{(3m^2-m+1)mn^2+m(11-8m)n-12m+m(n-1)(n-2)(n-3)+(n-1)N}\right. \\
&\times \log\frac{(m^2+m-1)n^2+2(5-2m)n-13}{(3m^2-m+1)mn^2+m(11-8m)n-12m+m(n-1)(n-2)(n-3)+(n-1)N} \\
&+ \frac{2[(m^2-m+1)n^2+2(1-m)n-1]}{(3m^2-m+1)mn^2+m(11-8m)n-12m+m(n-1)(n-2)(n-3)+(n-1)N} \\
&\times \log\frac{(m^2-m+1)n^2+2(1-m)n-1}{(3m^2-m+1)mn^2+m(11-8m)n-12m+m(n-1)(n-2)(n-3)+(n-1)N} \\
&+ \frac{(n-1)^2(n-3)}{(3m^2-m+1)mn^2+m(11-8m)n-12m+m(n-1)(n-2)(n-3)+(n-1)N} \\
&\left.\times \log\frac{(n-1)^2}{(3m^2-m+1)mn^2+m(11-8m)n-12m+m(n-1)(n-2)(n-3)+(n-1)N}\right\}
\end{aligned}$$

When $3 \le t \le \frac{n}{2} - 1$,

$$
\begin{aligned}
&H_{\mathrm{FB}}(t)\\
&= -m\Bigg\{\frac{(t-1)[(m^2+m-1)n^2+2(3-2m)n-5]}{[(t+1)m^2+(t-3)m-t+3]mn^2+(5t-3-4tm)mn-4m(t-1)+m(n-1)(n-2)(n-t-1)+(n-1)N}\\
&\times \log\frac{(m^2+m-1)n^2+2(3-2m)n-5}{[(t+1)m^2+(t-3)m-t+3]mn^2+(5t-3-4tm)mn-4m(t-1)+m(n-1)(n-2)(n-t-1)+(n-1)N}\\
&+\frac{2[(m^2-m+1)n^2+2(1-m)n-1]}{[(t+1)m^2+(t-3)m-t+3]mn^2+(5t-3-4tm)mn-4m(t-1)+m(n-1)(n-2)(n-t-1)+(n-1)N}\\
&\times \log\frac{(m^2-m+1)n^2+2(1-m)n-1}{[(t+1)m^2+(t-3)m-t+3]mn^2+(5t-3-4tm)mn-4m(t-1)+m(n-1)(n-2)(n-t-1)+(n-1)N}\\
&+\frac{(n-1)^2(n-\mathrm{t}-1)}{[(t+1)m^2+(t-3)m-t+3]mn^2+(5t-3-4tm)mn-4m(t-1)+m(n-1)(n-2)(n-t-1)+(n-1)N}\\
&\times \log\frac{(n-1)^2}{[(t+1)m^2+(t-3)m-t+3]mn^2+(5t-3-4tm)mn-4m(t-1)+m(n-1)(n-2)(n-t-1)+(n-1)N}\Bigg\}
\end{aligned}
$$

While $\frac{n}{2}-1<t\le\frac{n}{2}$ and *n* is an odd number,

$$
\begin{aligned}
&H_{\mathrm{FB}}(t)\\
&= -m\Bigg\{\frac{(t-1)[(m^2+m-1)n^2+2(3-2m)n-5]}{[(t+1)m^2+(t-3)m-t+3]mn^2+(5t-3-4tm)mn-4m(t-1)+m[(2m-1)n^2-(2m+1)n+2](n-t-1)+(n-1)N}\\
&\times \log\frac{(m^2+m-1)n^2+2(3-2m)n-5}{[(t+1)m^2+(t-3)m-t+3]mn^2+(5t-3-4tm)mn-4m(t-1)+m[(2m-1)n^2-(2m+1)n+2](n-t-1)+(n-1)N}\\
&+\frac{2[(m^2-m+1)n^2+2(1-m)n-1]}{[(t+1)m^2+(t-3)m-t+3]mn^2+(5t-3-4tm)mn-4m(t-1)+m[(2m-1)n^2-(2m+1)n+2](n-t-1)+(n-1)N}\\
&\times \log\frac{(m^2-m+1)n^2+2(1-m)n-1}{[(t+1)m^2+(t-3)m-t+3]mn^2+(5t-3-4tm)mn-4m(t-1)+m[(2m-1)n^2-(2m+1)n+2](n-t-1)+(n-1)N}\\
&+\frac{(n-\mathrm{t}-1)[(2m-1)n^2-2mn+1]}{[(t+1)m^2+(t-3)m-t+3]mn^2+(5t-3-4tm)mn-4m(t-1)+m[(2m-1)n^2-(2m+1)n+2](n-t-1)+(n-1)N}\\
&\times \log\frac{(2m-1)n^2-2mn+1}{[(t+1)m^2+(t-3)m-t+3]mn^2+(5t-3-4tm)mn-4m(t-1)+m[(2m-1)n^2-(2m+1)n+2](n-t-1)+(n-1)N}\Bigg\}
\end{aligned}
$$

While $\frac{n}{2}-1<t\le\frac{n}{2}$ and *n* is an even number,

$$
\begin{aligned}
&H_{\mathrm{FB}}(t)\\
&= -m\Bigg\{\frac{(t-1)[(2m-1)n^2+2(m-2)n-7]}{(t-1)(2m-1)mn^2+(t-1)(2m-5)mn-6m(t-1)+m[(2m-1)n^2-(2m+1)n+2](n-t+1)+(n-1)N}\\
&\times \log\frac{(2m-1)n^2+2(m-2)n-7}{(t-1)(2m-1)mn^2+(t-1)(2m-5)mn-6m(t-1)+m[(2m-1)n^2-(2m+1)n+2](n-t+1)+(n-1)N}\\
&+\frac{(n-\mathrm{t}+1)[(2m-1)n^2-2mn+1]}{(t-1)(2m-1)mn^2+(t-1)(2m-5)mn-6m(t-1)+m[(2m-1)n^2-(2m+1)n+2](n-t+1)+(n-1)N}\\
&\times \log\frac{(2m-1)n^2-2mn+1}{(t-1)(2m-1)mn^2+(t-1)(2m-5)mn-6m(t-1)+m[(2m-1)n^2-(2m+1)n+2](n-t+1)+(n-1)N}\Bigg\}
\end{aligned}
$$

S4. FBSE of caveman network in steady states

The task here is to determine at what *t* value the caveman network regains its non-heterogeneity. At evolutionary time *t*, the degree value sum of the inter-community edges in a community is $2\sum_{k_{\text{out}}}\theta_{k_{\text{out}},t}k_{\text{out}}$. For a given node *k*, if the maximum flow of any other node k' is independent of k, it must fulfill the condition

$$2\sum_{k_{\text{out}}}\theta_{k_{\text{out}},t}k_{\text{out}}-2\theta_{k,\text{out}}^{t}\geq d_{k'}.$$

This above inequality is satisfied for $\forall k$ and is equivalent to

$$2\sum_{k_{\text{out}}}\theta_{k_{\text{out}},t}k_{\text{out}}\geq\max_{k'}d_{k'}+\max_{k}2\theta_{k,\text{out}}^{t}$$

$$4t\geq 2(n-1)+4$$

$$t\geq\frac{n+1}{2}$$

where *n* is a positive integer. When the caveman network evolves into $\frac{n}{2}+1$ when *n* is an even number or $\frac{n+1}{2}$ when *n* is an odd number, the control of any node *k* on the maximum network flow only affects the nodes in set $\Omega=\{k'|a_{kk'}\neq 0,k'\neq k\}$. Here $a_{kk'}$ is the element at line *k* and column k' in adjacent matrix $\boldsymbol{A}$. At this *t* value the caveman network reaches its steady state. For $\forall k$,

$${}^{k}\boldsymbol{W}_{i,j}-{}^{k}\boldsymbol{W}_{i,j}^{*}=\begin{cases}1 & i\in\Omega\ \text{or}\ j\in\Omega\\ 0 & \text{others}\end{cases}$$

$$|\Omega|=n-1.$$

Thus,

$$b_k=\sum_{(i,j\in S(k))}\left({}^{k}\boldsymbol{W}_{i,j}-{}^{k}\boldsymbol{W}_{i,j}^{*}\right)\equiv 2(n-1)\cdot(nm-2)-\mathrm{C}_{n-1}^{2}$$

$$=(n-1)\cdot(2nm-\mathrm{n}-2).$$

The FBSE of the caveman network in s steady state ($\frac{n}{2}<t\leq n$) is

$$H_{\text{FB}}(t)=-m\sum_{k_{\text{out}}=0}^{2}\theta_{k_{\text{out}},t}\cdot\frac{b_{\arg k_{\text{out}},t}+d_{\arg k_{\text{out}},t}}{\sum_{q=1}^{N}(b_{q,t}+d_{q,t})}\log\frac{b_{\arg k_{\text{out}},t}+d_{\arg k_{\text{out}},t}}{\sum_{q=1}^{N}(b_{q,t}+d_{q,t})}=$$

$$-m\left\{\frac{[(2m-1)n^2-2mn+1]n}{[(2m-1)n^2-(2m+1)n+2]mn+(n-1)N}\times\log\frac{(2m-1)n^2-2mn+1}{[(2m-1)n^2-(2m+1)n+2]mn+(n-1)N}\right\}=\log N$$

where $b_{\arg k_{\text{out}},t}$ is the betweenness flow of a node with k_{out} inter-community edges at time *t*, $d_{\arg k_{\text{out}},t}$ is the degree value of a node *k* with k_{out} inter-community edges at time *t*, $b_{k,t}$ is the betweenness flow of node *k* at time *t*, and $d_{k,t}$ is the degree value of node *k* at time *t*.
